# Commencements

**Published:** 1890-08

**Authors:** 


### COMMENCEMENTS.

### University of Pennsylvania—Department of Dentistry.

At a public commencement held Thursday, May 1, 1890, at the American Academy of Music, the degree of Doctor of Dental Surgery was conferred by William Pepper, M.D., L.L.D., Provost, upon the following gentlemen, after which an address was delivered by J. William White, M.D., Professor of Clinical Surgery:

| NAME. | STATE. | NAME. | STATE. |
|---|---|---|---|
| Addicks, Hinrich, | Germany. | Hyatt, L. Sanford, | Pennsylvania. |
| Barnard, William E., | Maryland. | Hyndman, G. Edward, | Canada. |
| Bowman, Ira C., | Pennsylvania. | Jackson, Harvey N., | Wisconsin. |
| Burket, Frank S., | Pennsylvania. | Keener, George I., | West Virginia. |
| Chemlin, Wilhelm, | Germany. | Knight, Thomas B. P. | Pennsylvania. |
| Clement Fredrick G., | Pennsylvania. | Kusel, George C., | Pennsylvania. |
| Cohn, Alfred J., | Wisconsin. | Lane, James G., | Pennsylvania. |
| Compton, James D., | Pennsylvania. | Lankester, Frank J., | England. |
| Couto, Henrique S. de., | Brazil. | Leard, Norman W., | Prince Ed. Island. |
| Crothers, J. Ernest, | Ohio. | Leech, William W., | Pennsylvania. |
| Davenport, Kirk A., | New York. | Lohoff, Mortiz, | Germany. |
| Davenport, William S., | New York. | Lombard, Rafael M., | Cuba. |
| DeHaven, William W., | Pennsylvania. | Ludwick, John G., | Pennsylvania. |
| Dilk, Justino S., | Brazil. | McCartney, Frank M., | Pennsylvaia. |
| Doebbelin, Carl, | Germany. | Matten, Harry D., | Pennsylvania. |
| Drossel, Johann Otto, | Switzerland. | Norton, David A., | Wisconsin. |
| Dunn, William C., | Pennsylvania. | O'Hara, Patrick J., | Pennsylvania. |
| Eagan, Owen J., | Massachusetts. | Proctor, George S., | Pennsylvania. |
| Emerson, Charles A., | Ohio. | Pugh, Abe. L., | Pennsylvania. |
| Fair, Delhert D., | Illinois. | Requa, Herbert D., | New York. |
| Faye, Clement de., | Switzerland. | Romig, Edwin L., | Pennsylvania. |
| Faye, Edward de., | Switzerland. | Sacaza, Santiago, | Nicaragua. |
| Fenn, George L., | Connecticut. | Shennan, Lawson S., | Scotland. |
| Finlayson, A. Kay, | Scotland. | Snyder, Charles L., | Illinois. |
| Flotzinger, Aloysius, | Pennsyivania. | Spencer, Walter A., | Pennsylvania. |
| Freeman, Stewart, | Nova Scotia. | Stevens, Frederick W., | Nova Scotia. |
| French, Arthur T., | Florida. | Stewart, Howard T., | Mississippi. |
| Geilfuss, Edwin A., | Wisconsin. | Story, Horace B., | Ohio. |
| Girdwood, John, | Scotland. | Trout, Will H., | Pennsylvania. |
| Graves, Edwin D., | Pennsylvania. | Valiente, Francis P., | Cuba. |
| Haupt, J. A. Jacob, | Pennsylvania. | Whitmarsh, Herbert D., | New York. |
| Heitmuller, Karl, | Germany. | Williams, G. Frank, | Connecticut. |
| Holly, Sabourin, | Hayti. | Wood, Linneaus B., | Massachusetts. |

Total 66

DEGREE CONFERRED JUNE 5, 1889.

| | | | |
|---|---|---|---|
| Lowry, Samuel S., | Pennsylvania. | Ruis, Jesus, | U. S. of Columbia. |
| Ribas, Domingos P., | Brazil. | Sauers, Frederick, | Pennsylvania. |

The number of matriculates session 1889-90 ........ 159.

JAMES TRUMAN, Dean.

To Increase the Population.—A bill has been introduced into the French Senate to tax all bachelors. Perhaps the same remedy will be advocated here, as a recent census of the population of Fifth avenue, New York, showed about half a child to a family.

### College of Dental Surgery of the University of Michigan.

The fifteenth annual commencement of this department was held in University Hall, Ann Arbor, Thursday, June 26th, 1890. The number of matriculates was 106.

The degree of Doctor of Dental Surgery was conferred on the following candidates by the president of the university, James B. Angell, L.L.D.

William R. Calhoun, ............Mich.
Leo David Camp, ................Mich.
Ernest Catt, ...... ....England.
Charles Eli Collamer, ............Mich.
Charles Floyd Cook, ..............Mich.
George Howe Copp, ..............Mich.
Norman Kershaw Cox, New Zealand.
Charles Hugo Farman, ............Cal.
Fred. Dawson Fisher, .... ....... Eng.
Ida Gray, .................... ....Ohio
Jonn Jarius Green, .... ........Mich.
John Joseph Giusti, .......... .. Cal.
Bertrand Francois Hall, ........ Mich.
Edgar Allen Honey, .... ........Mich.
William George Howley, ........Mich.
Carolyn Murray McElroy, .......Ohio.
Melville Arthur Mason, ...........Ind.
Chester Cleveland Merriman, ...Wis.
John B. Keesing, Boston, .. ....Mass.
Eli Louis Moore, ............ Mich.
George Northcroft, ...........England.
Henry Turner Osborne, .............Ont.
James Andrew Oswald, .......... Mich.
Albert John Rust, ........New York.
Charles B. Scudder, ........ New York.
Alice Lovyse Sherman, ...... .. ..Wis.
William Hall Sieberst, .............Cal.
Fred. Cameron Sizelan, ....New York.
Mortimer F. Stever, ..........Iowa.
Fritz Bernhart Tegener, ....... Texas.
George T. Thuerer, .............. Wis.
Howard Devon Van Antwerp, .... Ky.
Gerrit Henry Veldhuis, ..........Mich.
John Hardin Waterhouse, .. .. Ohio.
Charles Elmer Welch, ............ Ind.
Gordon William Welch, .. .... Mich.
Harry Lloyd Williams, ......Mich.
Paul Woolsey, ...................Mich.

### Northwestern University.—Dental Department.

The first annual graduating exercises of the University Dental College, Dental Department of the Northwestern University, of Chicago, were held in connection with the medical department, at Central Music Hall, on April 29th, at 2:30 P. M.

The following named gentlemen received the degree of Doctor of Dental Surgery :

Isaac A. Freeman,
Arthur E. Matteson,
John B. Palmer,
Chas. W. Richardson,
William C. Wise,
Samnel H. Hunt,
William B. McCord,
Lucius E. Richardson,
William O. Vallette,
Sylvester M. Wilkie.

METALLIC NERVE POISONING.—Lead poisoning is essentially nerve poisoning, as indicated by its leading symptoms—pain and paralysis. Also in poisoning by arsenic and phosphorus, the phosphorised colloids of the nerves are precipitated, and in fatal cases the cause of death is a direct chemical combination of the poison with the component parts of the nerves.

### Boston Dental College.

The twenty-third annual commencement of this department was held in Berkley Temple, Wednesday, June 18th, 1890.

The number of matriculates was 80.

The degree of Doctor of Dental Surgery was conferred on the following graduates by the President of the University, I. J. Wetherbee, D.D.S.

| NAME. | RESIDENCE. |
|---|---|
| Herbert William Adams | Mass. |
| Walter Henry Arnold | Mass. |
| Wallace Bardeen | N. Y. |
| John Winslow Bailey | Mass. |
| George Franklin Beard | Mass. |
| George Wait Bosworth | Mass. |
| Alpheus Roberts Brown | Mass. |
| William Jamieson Currie | N. B. |
| Ernest Everett Gleason | Mass. |
| Charles Warren Hammett | Mass. |
| Patrick Ignatius Kelley | Mass. |
| William Nelson Kidder | N. H. |
| Edwin Fortis Lamson | Mass. |
| Lewis Eli Bringham Lansom | Mass. |
| John Henry Martin | Mass. |
| George Herbert Newland | Vt. |
| Edward John Palmer | Mass. |
| Benj. Archer Phillips | Mass. |
| Annie Felton Reynolds | Mass. |
| Ralph Frank Runals | Mass. |
| Frederick Albert Sawyer | Mass. |
| Charles William Tobin | Mass. |
| Cha les Pilkington Vesper | Mass. |
| Adolph Wengenroth | Mass. |
| Fred Murch Whitehouse | Maine. |
| Auville Leroy Whitney | Maine. |
| Sumner Parmenter Willard | Mass. |

### Harvard University—Dental Department.

*Dear Sir :*—On Wednesday, June 25th, the commencement exercises of Harvard University were held, as usual, in Sanders Hall at which the degrees were conferred upon the following dental students:

| NAME. | RESIDENCE. |
|---|---|
| S. R. Bartlett | Boston. |
| H. O. Dixon | Milford, N. H. |
| B. H. Codman | Boston. |
| E. H. Dixon | So. Eliot, Me. |
| A. W. Eldred | Worcester. |
| C. M. Keep | Brookline. |
| C. E. Luce | Walnut Hill. |
| Kotai Masuda | Tokio, Japan. |
| A. J. Oldham | Wellesley Hills. |
| H. Paal | Osnabruck, Ger. |
| C. E. Perkins | Brocton. |
| O. Pulvermacher | Berlin, Germany. |
| E. Rolfe | Valparaiso, Chili. |
| E. A. Shorey | Dover, N. H. |
| F. T. Taylor | New Bedford. |
| C. B. Tiscomb | San Francisco, Cal. |

Yours truly,

THOS. H. CHANDLER, Dean.

THE DOCTOR—"I think, my dear sir, that it would be better to have a consultation; your wife is seriously ill."

ANXIOUS HUSBAND—"There! I told my wife long ago she ought to have proper medical advice, but she was so afraid of hurting your feelings."

